# Longitudinal associations of psychosocial factors and fear of falling in older adults: a systematic review

**DOI:** 10.1186/s12877-026-07463-1

**Published:** 2026-04-29

**Authors:** Laura Jesgarz, Moritz Gehring, Sarah K. Schäfer, Susanne Wurm

**Affiliations:** 1 Department of Prevention Research and Social Medicine, Institute for Community Medicine, University Medicine Greifswald, Walther-Rathenau-Straße 48, Greifswald, 17475 Germany; 2 Leibniz Institute for Resilience Research, Mainz, Germany; 3 Department of Clinical Psychology and Psychotherapy for Children and Adolescents, Technische Universität Braunschweig, Braunschweig, Germany; 4 Department of Clinical Child and Adolescent Psychology and Psychotherapy, RPTU University Kaiserslautern-Landau, Landau, Germany

**Keywords:** Fear of falling, Psychosocial, Incidence, Longitudinal, Older people

## Abstract

**Background:**

Fear of falling (FOF) is a phenomenon prevalent among older adults associated with adverse outcomes, including reduced mental and physical health, decreased quality of life, and an overall reduction in social participation. Based on cross-sectional and longitudinal studies, recent reviews have provided information on the prevalence of FOF and its association with different health outcomes. To gain a deeper understanding of factors relevant to interventions to prevent or reduce FOF, this review goes beyond previous evidence syntheses by focusing on psychosocial factors that are longitudinally associated with FOF. The objective of the review is to provide an overview on evidence levels of psychosocial factors assumed to be longitudinally associated with FOF.

**Methods:**

Studies examining associations between psychosocial factors (predictors) and FOF (outcome) longitudinally were included. Cochrane CENTRAL, Embase, Scopus, and Web of Science were searched up to October 25, 2025. An ordinal rating scheme was used for data synthesis to assess beyond sociodemographic variables and other psychosocial factors. A modified version of the Newcastle–Ottawa scale was employed to evaluate study quality.

**Results:**

Sixteen studies (*n* at baseline: 255–9,060) with a total of 30,724 participants reported longitudinal associations of 14 psychosocial factors with FOF. Higher depressive symptoms, anxiety, negative affect, and symptom burden, lower social activity, social participation, emotional support, and feeling older were linked to higher FOF. The relationship between depressive symptoms and higher FOF showed the most robust evidence, with six studies finding evidence for incremental validity of depressive symptoms beyond sociodemographic variables and other psychosocial factors. In contrast, higher self-efficacy, positive affect, social support, and social cohesion were associated with lower FOF. In some cases, the magnitude of associations was reduced when controlling for other variables.

**Conclusion:**

The evidence base remained weak for psychosocial factors other than depressive symptoms. Further longitudinal research is needed on the role of psychosocial factors for FOF. Such studies enlarge the evidence base for factors identified in this review and should include additional factors (e.g., loneliness). Our findings highlight the need for further research on the relationship between depressive symptoms and FOF for the development of effective interventions.

**Trial registration:**

Pre-registration ID: https://doi.org/10.17605/OSF.IO/X5ZGR

**Supplementary Information:**

The online version contains supplementary material available at 10.1186/s12877-026-07463-1.

## Background

Fear of falling (FOF) is a psychological construct that was initially described as a phobic reaction to standing or walking after a fall, called ‘ptophobia’ [5]. In recent research, the conceptual framework of FOF has undergone an expansion, integrating reductions in balance self-efficacy [69, 71] to avoid falls during everyday activities. However, excessive concerns may manifest as an anxiety disorder, with which they share several characteristics, including anxious cognitions or marked avoidance [15, 53]. Anxious cognitions in FOF include negative thoughts and beliefs (e.g., low balance self-related), and heightened attention to instability, creating a vicious cycle where fear leads to cautious behaviors (e.g., shuffling, grabbing objects) that actually increase fall risk by reducing physical activity [1]. Within the Diagnosis and Statistical Manual of Mental Disorders (DSM-5), the specific phobia section explicitly addresses fear of falling among older adults since 2013 [16].

In a systematic review of 153 studies on older adults, Xiong et al. [82] reported heterogeneous prevalence estimates for FOF ranging from 6.96 to 90.34%. FOF can result in various negative health outcomes, including future falls, higher mortality, comorbidities, limitations in daily activities, mobility restrictions, and higher frailty, a decrease in muscle strength, physical capacity, objective and subjective well-being, and lower life satisfaction [3, 20, 32, 60, 76]. In order to develop effective interventions for the prevention and treatment of FOF, it is crucial to gain a comprehensive understanding of the psychosocial factors that contribute to the development and persistence of FOF. The systematic review by Xiong et al. [82] identified 28 risk factors being significantly associated with FOF. These include sociodemographic characteristics such as female gender, older age, low educational level, living alone, and a history of falls [7, 13, 68, 77, 82]. In addition, health-related aspects were identified as potential risk factors for FOF: higher BMI, lower physical function, the use of walking aids, frailty status, poor perceived health, lower scores of muscle strength, joint function, and balance [10, 23, 43, 67, 82]. Furthermore, health impairments such as hip fracture, knee osteoarthritis, hearing impairment, visual impairment, and body pain have been found to be risk factors for FOF [31, 68, 75, 82].

A scoping review [41] summarized studies on factors that are associated with FOF published between 2015 and 2020, found that the majority of studies investigated FOF cross-sectionally (30 studies), while a smaller number examined longitudinal associations (14 studies). The findings for psychosocial factors indicated that anxiety and depressive symptoms were most often analyzed (20 studies) and most consistently associated with FOF (19 studies). The majority of studies (12 studies) investigated the relationship between depressive symptoms and FOF cross-sectionally (e.g., [11, 28, 43, 51, 57]) while five studies reported on longitudinal associations (e.g., [17, 54, 72]). One study reported an association between lower levels of social support and FOF [43], while Dierking et al. [17] reported a relationship between the number of family members seen monthly and lower FOF. Hajek et al. [25] found that various psychological factors, such as higher levels of loneliness, higher negative affect, lower life satisfaction, lower positive affect, as well as lower levels of optimism, self-efficacy, self-esteem, self-regulation, and more perceived stress, were cross-sectionally associated with higher FOF. Furthermore, individual studies found an association cross-sectionally between lower life satisfaction and FOF [46] as well as an association between lower morale and higher FOF [52]. Taken together, the vast majority of studies on psychosocial factors and FOF are based on cross-sectional data, whereas comparatively little is known about the respective longitudinal associations.

The objective of this systematic review is therefore to present a comprehensive overview of psychosocial risk and protective factors that are longitudinally associated with FOF. The following research questions were addressed in the review:

1. Which psychosocial factors are longitudinally examined in relation to FOF?
2. Which psychosocial factors are longitudinally associated with FOF in older adults?
3. What is the level of evidence for specific psychosocial factors associated with FOF in older adults?
4. To what extent do associations between specific psychosocial factors and FOF demonstrate incremental validity of these factors beyond (1) sociodemographic and (2) other psychosocial factors?

In contrast to previous reviews [41, 82], our evidence synthesis is thus focused on longitudinal studies examining the association of psychosocial factors and FOF. Although simple longitudinal associations do not allow for causal inference, this methodological approach has the advantage of offering a more nuanced understanding of the relationship between psychosocial factors as predictors and FOF as an outcome. Moreover, we focus not only on the bivariate association between psychosocial factors and FOF but also study their incremental validity beyond sociodemographic variables and other psychosocial factors. In so doing, we derive knowledge on the unique value of specific psychosocial factors. Based on these findings, we aim at providing new insights for prevention and treatment strategies and identify potential areas for further research.

## Method

The systematic review is reported in line with the PRISMA guidelines [49]. The review protocol was prospectively preregistered at the Open Science Framework (preregistration. ID: 10.17605/OSF.IO/X5ZGR). All deviations from the review protocol are documented in Supplementary Material 1. The research questions in particular were further differentiated, and the aspects of interest were addressed in three additional questions.

### Search strategy

The search was conducted on November 18, 2023, and lastly updated on October 25, 2025, using six electronic databases selected for their relevance to the research question (i.e., APA PsycNET, CINAHL, Cochrane CENTRAL, Embase, Scopus, and Web of Science). The search terms comprised four clusters: (1) population (e.g., "older adults" OR "old* people" OR "aging"); (2) exposure (e.g., "fear AND falling" OR FOF OR "fall-efficacy"); (3) psychosocial factor (e.g., factor* OR predict* OR correlate*); and (4) study design (e.g., longitudinal OR "long-term" OR wave OR "follow-up"). MeSH/Emtree terms were used where applicable. The terms within each cluster were connected using the Boolean operator OR, and the clusters were linked using the operator AND. The full search strategy for each database is presented in the Supplementary Material 2. The electronic database search was amended on July 15, 2024, by a manual search in the reference lists of the included primary studies and related systematic reviews [41, 82]. For maximum sensitivity, we searched for studies citing those studies included in our review via Google Scholar on July 19, 2024.

### Inclusion and exclusion criteria

The studies included were required to examine a population of older adults aged 65 or older living in a private household, regardless of the subjects’ physical and mental health status, gender, geographic location, ethnicity, and nationality. The decision to concentrate on community-dwelling older adults was made because older adults who are living in residential care facilities or nursing homes potentially differ in many ways from those living in private households, which in consequence affects the relationship between psychosocial factors and FOF. In particular, older adults in residential care facilities or nursing homes clearly differ in their age, physical and cognitive health, accompanied by heightened care requirements, frailty [38], or sarcopenia [50]. For example, a study that was based on German health insurance claims data showed a prevalence of dementia of 51.8% in nursing home residents, opposed to 2.7% in community-dwelling older adults [29]. In addition, a multitude of environmental factors differ between older people living in private households versus long-term care settings. Consequently, FOF in this population may be impacted by additional predictor variables, and also the development of interventions for this target group may require different approaches.

The main focus is the association of a variety of psychosocial factors and FOF, whereby FOF represents the outcome variable. Outcome operationalizations were not defined in advance.

The term “psychosocial factors” as used in the context of this review includes those factors relating to psychological factors (i.e., depressive symptoms) as well as social factors such as social support, social integration, or social activities.

Studies were excluded if the psychosocial factors were not treated as independent variables/predictors^1^ in regression models, and if FOF was not considered as the outcome. Studies limiting their analyses to cross-sectional associations between psychosocial factors and FOF were excluded. Moreover, all studies that were not primary research reporting on empirical data (such as research protocols, theoretical papers) were excluded.

### Study selection

Following deduplication in *Zotero*, the studies were reviewed by two independent members of the review team (LJ, MG) in *Rayyan* [48] at the full-text level. The interrater reliability at the title/abstract level was moderate (*kappa* = 0.58), and at the full-text level it was good (*kappa* = 0.70). Disagreements were resolved through discussion or by consulting supervisory members of the review team (SKS, SW).

### Data extraction

A suitable data extraction sheet was developed for the purpose of this review. The data from the primary studies included were extracted by one reviewer (LJ) and checked by a second (MG). Extracted information included characteristics of the sample, study design, the assessment of FOF, information on psychosocial factors and their assessment, as well as information on statistical models employed to examine the link between psychosocial factors and FOF. Moreover, the data extraction sheet included the rating scheme developed to assess the predictive value of psychosocial factors (see [59]).

### Quality appraisal

The quality of primary studies was examined as an indicator of the potential risk of bias. This was done by employing a modified version of the Newcastle–Ottawa Scale (NOS; 79), which was utilized to identify risk of bias related to 1) sample selection, 2) comparability, 3) outcome, and 4) analysis. The assessment was conducted by two independent research team members (LJ, MG). Conflicts were discussed and resolved between the two team members. The NOS was modified to assess relevant aspects that contributed to a better quality of the studies included, i.e., the use of a standardized measurement for FOF, the documentation and analysis of losses to follow-up, as well as the justification and modelling of confounders. The modified version of the NOS was uploaded to OSF prior to the start of the quality assessment. The total scores were calculated based on single items, with possible values ranging from 0 to 15, representing the overall quality rating of each study. The median and mean of the total scores were calculated to provide an overall estimate of the quality of evidence.

### Data synthesis

To examine the predictive value of psychosocial factors for FOF outcomes, we adapted a rating scheme that was recently developed to assess the predictive value of resilience factors for mental responses to stress [59]. This rating scheme was used to examine both the direction and the consistency of findings reported across primary studies and distinguishes between nine different levels of evidence (see Table 1).

**Table 1:**
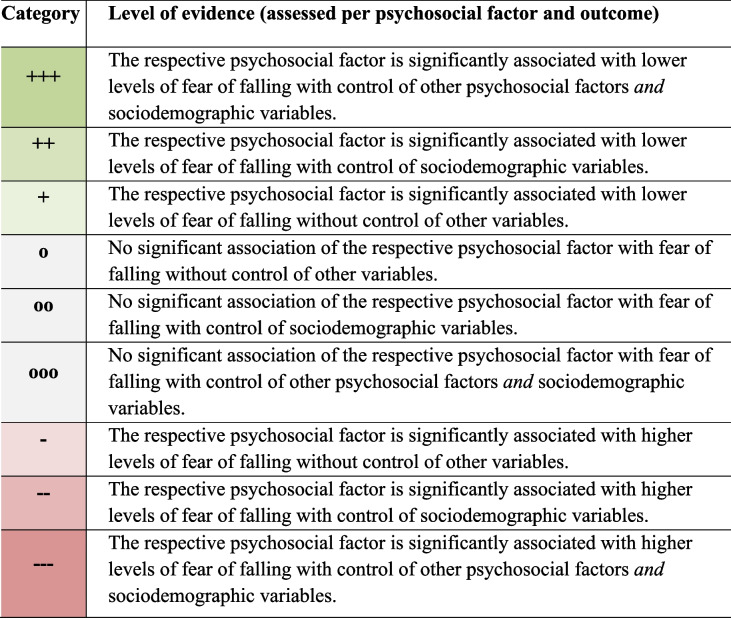
Rating of evidence levels of psychosocial factors

Evidence levels ranging from + + + (= most favorable level of evidence for the respective psychosocial factor from a single study, i.e., the respective psychosocial factor showed incremental validity beyond other psychosocial factors with the highest level of control for other variables) to --- (= least favorable level of evidence for the respective psychosocial factor from a single study, i.e., there is evidence for the respective psychosocial factor being associated with higher levels of FOF with the highest level of control for other variables)

In cases where coding in primary studies was inverse, that is, higher scores indicated lower levels of the respective psychosocial factor (e.g., poor social support), the respective coding was inverted for our rating scheme in a way that higher scores indicated higher levels of the respective psychosocial factor

The synthesis of findings across studies is challenging as single factors are examined in heterogeneous regression models, including different types and numbers of predictor variables, with low-between study homogeneity of predictor variables. Thus, standard meta-analysis would risk producing biased results [56]. At the same time, solely qualitative syntheses provide little evidence on incremental validity and evidence levels per factor. Hence, a rating scheme was developed aiming to derive evidence ratings for each psychosocial factor that allow for between-factor comparisons.

Ratings were conducted for each psychosocial factor and its association with FOF. The model with the highest level of control (i.e., including the largest number of control and confounding variables) was used for this purpose, while other models were only examined for qualitative synthesis. Ratings were then categorized into three levels of evidence (see Table 1): The respective psychosocial factor showed a significant association with FOF, 1) without control of any other variable (+ or -); 2) with control of sociodemographic variables only (+ + or --); and 3) controlling of sociodemographic variables *and* other psychosocial factors (+ + + or ---). Positive ratings (+ to + + +) indicated that higher levels of the respective factor were associated with lower levels of FOF, while negative ratings (- to ---) indicated that higher levels of the respective factors were linked to higher levels of FOF. Ratings of + + + or --- provided the strongest evidence for incremental validity, with the psychosocial factor being associated with outcomes with the highest level of control for other variables.

In cases where primary studies identified null effects (i.e., non-significant) for the association of the psychosocial factor and FOF, a distinction was made as to whether the null effect was observed without control of any other variable (o), with control of sociodemographic variables (oo), or when both sociodemographic variables *and* other psychosocial factors were accounted for (ooo).

## Results

### Search outcomes

**Fig. 1:**
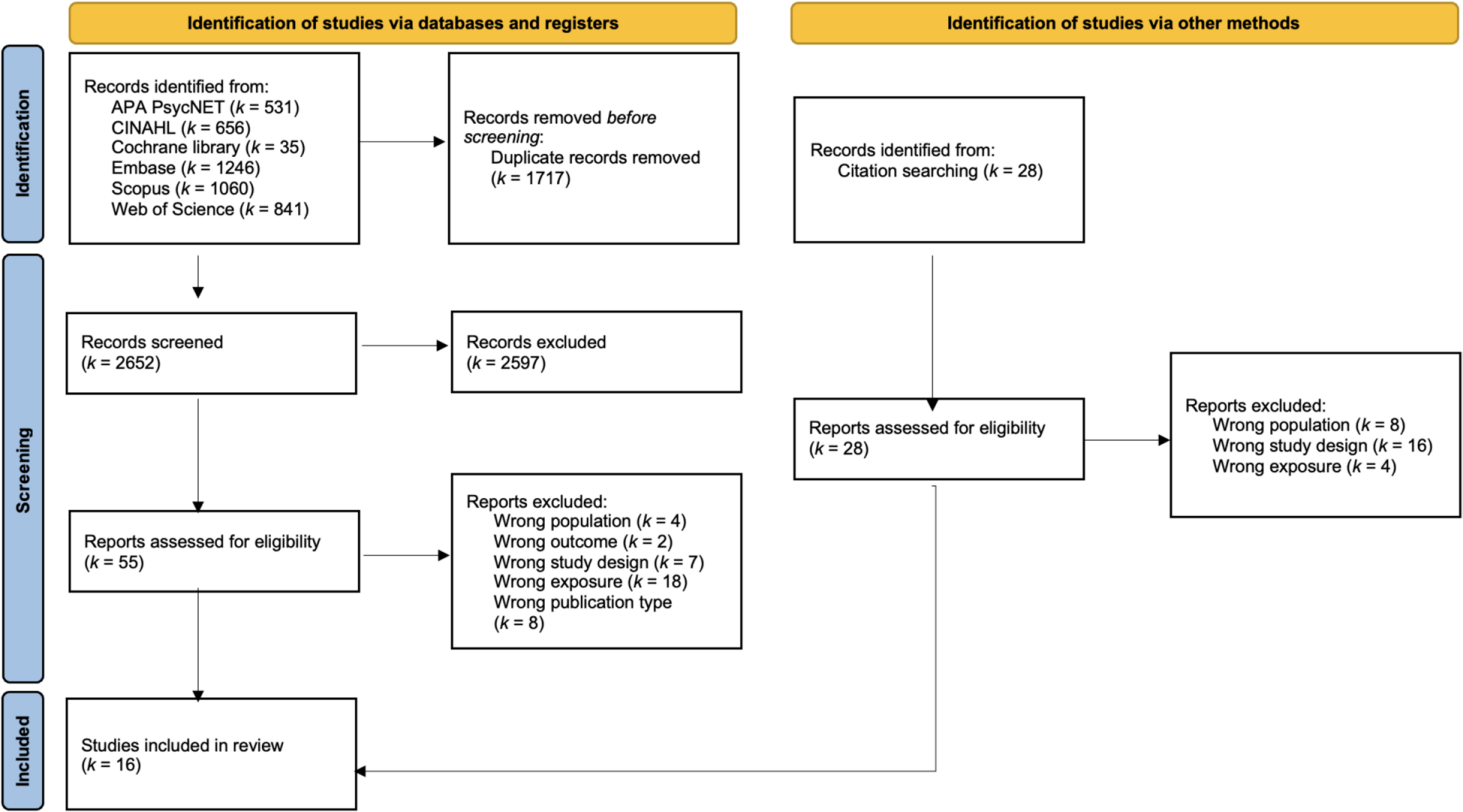
Flowchart in accordance with the Preferred Reporting Items for Systematic Reviews and Meta-Analyses (PRISMA) guidelines [49] (According to the Cochrane Handbook we used ‘n’ for the samples within primary studies and ‘k’ for the number of studies included in the review [27])

The search of the databases returned 2,675 hits. Of these, 893 were removed as duplicates. The remaining 1,782 records underwent initial screening at the title and abstract level, after which 53 records were subjected to examination at the full-text level. Following the exclusion of 37 records, a total of 16 studies were included in our synthesis (see Fig. 1). The search update on October 25, 2025, did not result in additional eligible primary studies.

### Study and sample characteristic

The sample sizes of primary studies ranged from 255 to 9,060 participants at baseline, with a total of 30,724 participants across all studies. The age range was 65–98 years. Furthermore, the proportion of female participants ranged from 35.1% to 100%. The majority of studies were conducted in the US [17, 22, 35, 40, 44, 47, 55], followed by Japan [42, 72, 78], Australia [4, 12], Sweden [37], Canada [13], Brazil [19], and different European countries [21]. The 16 studies included three types of FOF outcomes: onset (12), persistence (4), and changes in fear of falling (2).

A total of 14 studies were found to be population-based, one study used a convenience sample, and one study used a community-based sample. Fourteen studies focused on community-dwelling older adults; the majority examined both genders/sexes, and two studies included female participants only. Another study examined community-dwelling older adults who had experienced a fall before the baseline assessment and during the follow-up period. Finally, one study included exclusively older people suffering from Parkinson’s disease.

The attrition rate between baseline and follow-up assessments was reported in six out of 16 studies, with values ranging from 11.8% to 53.1%.

To measure FOF, one study used the Falls Efficacy Scale-International [83], another study used the Falls Efficacy Scale-Brazil [8], and one used a combination of the Falls Efficacy Scale-International [83] amended by a single question if the person is afraid of falling. The remaining 13 studies employed between one and three self-developed questions to assess FOF, e.g., ‘How afraid are you of falling?’. The response formats ranged from binary yes/no items to 3- or 4-point ordinal Likert scales with verbal anchors at each point. In several studies (e.g., [19, 37, 72]) scale scores were subsequently dichotomized for analysis, contrasting individuals with and without FOF. However, not all studies reported the exact wording of the questions or the exact response formats (see supplementary material 3 for details).

All studies employed different types of regression-based models for studying the link between psychosocial factors and FOF. The majority of the studies (*k* = 10) employed logistic regression models, while two studies each used Cox regression (*k* = 2) and binomial regression (*k* = 2). Single studies employed Poisson regression (*k* = 1) and linear regression (*k* = 1).

The included studies adjusted for a range of covariates, which can be categorized into sociodemographic factors and FOF-related variables. The most frequently controlled sociodemographic variables included age (*k* = 7) and gender (*k* = 7), followed by educational level (*k* = 6), income (*k* = 3), ethnicity (*k* = 4), and living situation (e.g., living alone; *k* = 4). In addition, several studies controlled for health-related and functional factors, in particular history of falls (*k* = 6), limitations in basic and instrumental activities of daily living (*k* = 5), chronic diseases (*k* = 5), cognitive function (K = 5), body mass index (*k* = 4), and self-rated health (*k* = 3). Other variables considered included pain (*k* = 3), mobility and balance problems (*k* = 3), as well as hospitalizations (*k* = 2) (Table 2).

**Table 2:** Characteristics of included primary studies

Study ID/Country	Population	Age M (SD) & range	Sample size	Outcome (FOF)	Validated assessment of outcome	Assessment waves	Psychosocial factors	Effect estimate presented	Risk of bias
Austin 2007 [4] Australia	Community-dwelling women, 100% female	75.2 (NR) NR	Baseline: 1,282 Follow-up: 1,282	Onset and Persistent FOF	No	Baseline, 3- years Follow-up	Depressive symptoms	OR = 2.58, 95% CI [1.56; 4.28]	High
Clemson 2015 [12] Australia	65- years or older community-dwelling, 53.3% female	73.4 (NR) 65–94	Baseline & Follow-up: 855	Onset FOF	No	Baseline: 1994 Follow-up: every 2 years for 11 years	Depressive symptoms	HR = 1.24, *p* =.000	Moderate
							Reduced social activity	HR = 1.81, *p* =.002	
							Positive affect	HR = 0.90, *p* =.003 (single predictor)	
							Negative affect	HR = 1.09, *p* =.007 (single predictor)	
Curcio 2020 [13] Canada, Albania, Brazil, Colombia	Community- dwelling older adults 52.5% female	NR (NR) 65–74	Baseline: 1,424 Follow-up: 1,409	Onset FOF	Yes	Baseline: 2014 Follow-up: 2016	Depressive symptoms	OR = 1.77, 95% CI [1.21; 2.59]	Moderate
Dierking 2016 [17] USA	Community-dwelling residents, 64.4% female	79.4 (NR) NR	Baseline: 1,682 Follow-up: 1,079	Onset FOF	No	Baseline: 2000–2001, Follow-up: 2004–2005; 2007–2008; 2010–2011	Social support: ‚Having someone to count on’ ‘Number on friends seen monthly’ ‘Number on family members seen monthly’	OR = 1.25; 95% CI [.90; 1.74] OR =.98; 95% CI [.95; 1.02] OR = 1.03; 95% CI [1.03; 1.06]	Moderate
							Depressive symptoms	OR = 1.02, 95% CI [1.03; 1.04]	
Drummond 2020 [19] Brazil	Community- dwelling older adults, residents of the north zone of Rio de Janeiro, 72% female	NR (NR) NR	Baseline: 742 Follow-up: 393	Onset and Persistent FOF	Yes	Baseline: 2009 Follow-up: 2013	Depressive symptoms	Onset: RR = 1.46 95% CI [0.75; 2.85] Persistent: RR = 1.50, 95% CI [1.08; 2.08]	Moderate
							Activity level	Onset: RR = 2.32, 95% CI [1.40; 3.07] Persistent: RR = 1.17, 95% CI [0.94;1.46]	
Freiberger 2022 [21] Europe	Community-dwelling people reporting a fall at baseline and follow-up, 54.2% female	79 (6.0) NR	Baseline: 389 Follow-up: 389	Onset and Persistent FOF	No	Baseline: 2004–2008 Follow-up: 2009	Depressive symptoms	OR = 3.54, 95% CI [1.23; 10.1]	High
Fundenberger 2022 [22] USA	US adults aged 65 and older 58.9% female	75.24 (6.75) NR	Baseline: 2,172 Follow-up: 1,679	Onset FOF	No	Baseline: 2011 Follow-up: 2017	Subjective aging	OR = 1.24 95% CI [1.08; 1.42]	High
							Depressive symptoms	NR	
Lach 2005 [35] USA	Community-dwelling residents, 60% female	75 (6.2) NR	Baseline: 890 Follow-up I: 842 Follow-up II: 600	Onset FOF	No	Baseline: Wave 3,4 Year: NR	Depressive symptoms	NR	High
							Social activity	OR = 0.58, 95% CI [0.17; 1.98]	
							Activity level	OR = 0.53, 95% CI [0.24; 1.17]	
Lindh-Rengifo 2019 [37] Sweden	Community-dwelling older adults with Parkinson 35.1% female	68 (9) NR	Baseline: 255 Follow-up: 165	Change of FOF	Yes	Baseline: 2013 Follow-up: 2016	Self-efficacy	B = −0.811 95% CI [−1.15; −0.466], β = −0.357	Moderate
							Depressive symptoms	B = 0.595 95% CI [−0.025; 1.22], β = 0.595	
Luo 2022 [40] USA	Sample of medicare beneficiaries aged 65 and older 58.1% female	78 (7.73) NR	Baseline: 6397 Follow-up: 6,376	Change of FOF	No	Baseline: 2015 Follow-up: 2016	Anxiety	RRR = 1.33, 95% CI [1.02;1.72]	High
							Depressive symptoms	RRR = 1.18; 95% CI [0.89; 1.57]	
Makino 2021 [42] Japan	Community-dwelling older adults 51.6% female	71.1 (4.7) NR	Baseline: 5,104 Follow-up: 2,469	Onset FOF	No	Baseline: 2011/12 Follow-up: 2015/16	Depressive symptoms	OR = 1.10; 95% CI [1.04; 1.16]	Moderate
Murphy 2003 [44] USA	Community-living older women	79.3 (5) 72–98	Baseline: 804 Follow-up: 313	Onset FOF	No	Baseline: 1989 Follow-up: 1990	Anxiety	RR = 1.41, 95% CI [.97; 2.05]	Moderate
							Depressive symptoms	NR	
							Emotional support	RR = 2.64, 95% CI [1.57; 4.41]	
Oh-Park 2011 [47] USA	Community- dwelling older adults Female: 53.1%	80 (5.24) NR	Baseline: 555 Follow-up: 380	Onset FOF	No	Baseline: 2004- 2008 Follow-up: 2009	Depressive symptoms	Onset: HR = 1.16 95% CI [1.07; 1.26] Persistent: HR = 1.45 95% CI [1.21; 1.74]	Moderate
Peng 2024 [55] USA	Community- dwelling older adults 58.4% female	76.5 (NR) NR	Baseline: 9,060 Follow-up: 9,060	Onset FOF	No	Baseline: 2011–2017 Follow-up: 2012–2018	Symptom burden	AOR = 1.18 95% CI [1.15; 1.22]	Moderate
Uemura 2015 [72] Japan	Community- dwelling older adults 37.9% female	70.8 (4.7) NR	Baseline: 1,929 Follow-up: 1,700	Onset FOF	No	Baseline: 2011/12 Follow-up 15 month later	Depressive symptoms	OR = 1.11; 95% CI [1.04; 1.15]	Moderate
Wang 2023 [78] Japan	Community- dwelling older adults 56.4% female	74.8 (7.1) NR	Baseline: 4,957 Follow-up I: 3,567 Follow-up II: 3,255 Follow-up III: 2,545	Onset FOF	No	Baseline: 2010 Follow-up: 2013, 2016, 2020	Social cohesion	OR = 0.82; 95% CI [0.71; 0.95]	High
							Social participation	OR = 1.17; 95% CI [1.07; 1.28]	
							Reciprocity	OR = 1.05 95% CI [0.62; 1.78]	

### Quality appraisal

**Fig. 2:**
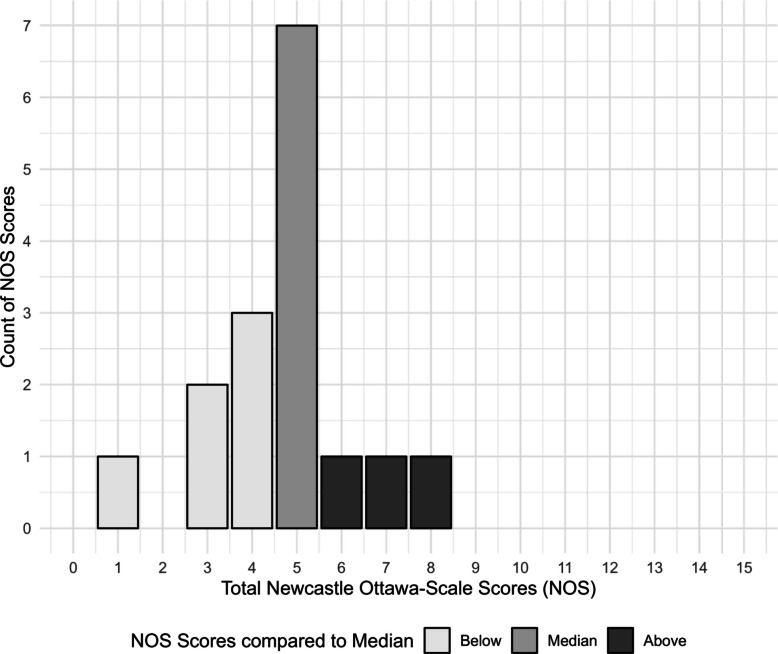
Quality appraisal of the primary studies included. Results of the study quality assessment conducted using the modified version of the Newcastle–Ottawa Scale [79]. Results are presented for 16 primary studies

Overall, study quality as indicated by the modified NOS was low to moderate, with a median quality score of 5 (*M* = 4.7, *SD* = 1.6, *Range* = 1–8). Risk of bias mostly resulted from low reporting standards (e.g., no reports on attrition; *k* = 13). Only one study analyzed differences between responders and non-responders with respect to variables of interest [19]. The majority of studies employed a non-standardized assessment for FOF (*k* = 13), failed to document (*k* = 16), and control for potential confounders (*k* = 15), and all drop-out rates were above 5%. Power analyses were only performed in one study [37] (Fig. 2).

With respect to research question 1 (*Which psychosocial factors were longitudinally examined in relation to FOF?*) the findings show that the association with depressive symptoms was examined most frequently (*k* = 14), followed by anxiety symptoms (*k* = 2), positive and negative affect, self-efficacy, symptom burden (*k* = 1), social activity and activity level (*k* = 2), social support, social participation, subjective age, emotional support, social cohesion, and reciprocity (*k* = 1).

### Synthesis

The following findings address research questions 2 to 4, that is, the psychosocial factors longitudinally associated with FOF in older adults, the level of evidence for these factors, as well as their incremental validity beyond sociodemographic and other psychosocial factors.

**Fig. 3:**
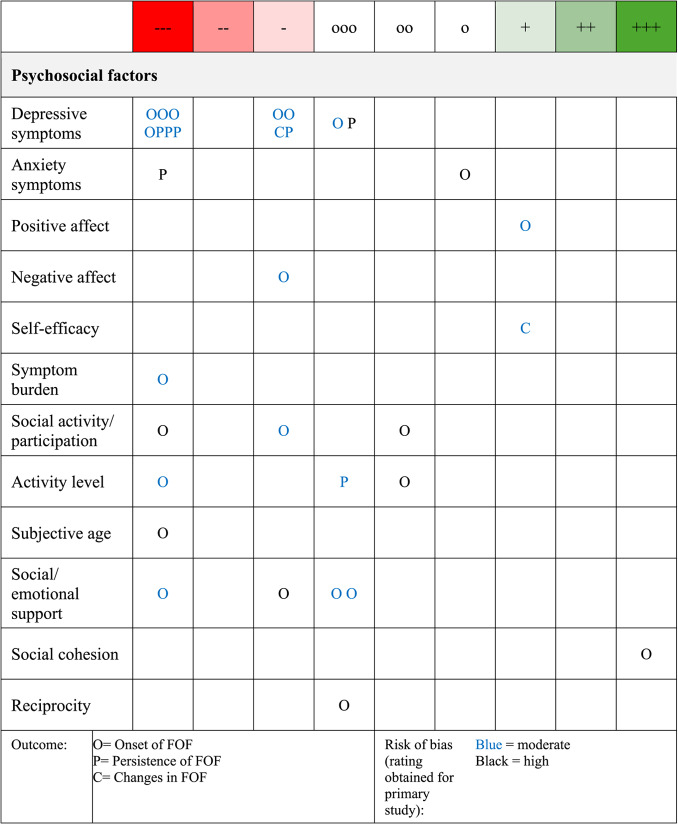
Evidence ratings per psychosocial factor. Evidence rating per psychosocial factor and its association with FOF. The letters indicate the operationalization of the outcome (Onset of FOF, Persistent FOF, or Changes in FOF), and colors represent the quality of the study for the current effect estimate (e.g., 4 effect estimates reported a link between depressive symptoms and the onset of FOF, while one effect estimate showed an association between depressive symptoms and persistence of FOF. The risk of bias in these studies was moderate for 5 effect estimates and high for 2 effect estimates). Evidence levels range from + + + (= most favorable level of evidence for the respective psychosocial factor from a single study, i.e., the respective psychosocial factor showed incremental validity of *lower* levels of FOF beyond other psychosocial factors with the highest level of control for other variables) to --- (= least favorable level of evidence for the respective psychosocial factor from a single study, i.e., there is evidence for the respective psychosocial factor being associated with *higher* levels of FOF with the highest level of control for other variables). Rating categories o, oo, and ooo represent statistically non-significant findings with different levels of control. See Table 1 for details on the rating scheme

Overall, a total of 32 effect estimates were reported across 16 primary studies (see Fig. 3). The effect estimates were distributed across evidence levels as follows: 12 effect estimates received a rating of ---, indicating strong evidence of the association of the psychosocial factor and a higher level of FOF under the highest level of control of other variables; 7 effect estimates received a rating of -, indicating limited support for the association without controlling for other variables. Three effect estimates received a rating of + + +, indicating strong evidence for the association of the psychosocial factor and a lower level of FOF when the highest level of control was applied. Two effect estimates received a rating of +, providing limited support for the association without controlling for other variables. Nine effect estimates were statistically insignificant (o to ooo).

The predominant focus of analysis was the relationship between depressive symptoms and FOF. The majority of these studies (*k* = 8) examined the relationship between depressive symptoms and the onset of FOF, of which four effect estimates suggested that depressive symptoms were associated with increased odds or hazards for developing FOF above and beyond other psychosocial factors and sociodemographic variables (---).^2^ Four effect estimates indicated that depressive symptoms were linked to an increased risk of developing FOF without control for other variables (-),^3^ and one effect estimate did not find a significant link between the variables (ooo; RR = 1.46; 95% CI [0.75; 2.85]); [19]).

In addition, studies considering the persistence of FOF (*k* = 5) showed that for three of the five studies, depressive symptoms were associated with an increased risk of having FOF above and beyond other psychosocial factors and sociodemographic variables (---)^4^; one effect estimate indicated an increased risk for FOF without control of other variables (-; OR = 2.58; 95% CI [1.56; 4.28]; [4]) and one study did not find a relationship between the variables (ooo; RRR = 1.18; 95% CI [0.89; 1.57]; [40]). Finally, one study examined the link between depressive symptoms and FOF over time, reporting that increased depressive symptoms were not associated with higher FOF scores at 3-year follow-up when controlling for other psychosocial factors, demographic variables, as well as gait- and mobility-related parameters (ooo; B = 0.595; 95% CI [−0.025; 1.22]; [37]).

The role of anxiety symptoms for FOF was examined in two studies, one of which analyzed the relationship to the onset of FOF [44] and reported an elevated risk for developing FOF in persons with higher anxiety symptoms, but not significant and without controlling for other factors (o; RR = 1.41; 95% CI [0.97; 2.05]). The other study investigated the relationship between anxiety symptoms and FOF levels at follow-up, indicating that persons with higher anxiety symptoms at baseline are subject to an increased risk of FOF at follow-up compared to persons without the presence of anxiety symptoms at baseline even after adjusting for other psychosocial factors and sociodemographic variables (---; RRR = 1.33; 95% CI [1.02; 1.72]; [40]). Furthermore, one study examined the association of both positive and negative affect with the onset of FOF [12]. One effect estimate indicated that positive affect was linked to a lower hazard of acquiring FOF across eleven years (+; HR = 0.90, *p* = 0.003), while negative affect was associated with a higher hazard for acquiring FOF during the same interval (-; HR = 1.09, *p* = 0.007). Hazard ratios for both predictors were reported without controlling for other variables, and both positive and negative affect were not included in the final cox regression models since they were removed after stepwise exclusion of variables not meeting the significance level of *p* < 0.01. Results referring to the role of self-efficacy were limited to findings from univariable linear regression for general self-efficacy at baseline and FOF at 3-year follow-up, suggesting that an increase in self-efficacy scores was univariably associated with reduced FOF scores at 3-year follow-up, but without controlling for other variables (+; *B* = −0.811, 95% CI [−1.15; −0.466], β = −0.357; [37]).

One study examined symptom burden and its relation to FOF at 1-year follow-up, revealing that higher symptom burden scores were linked to increased odds of FOF at 1-year follow-up after controlling for other psychosocial factors and sociodemographic variables (---; OR = 1.16, 95% CI [1.12; 1.20]; [55]).

Furthermore, three studies examined the association between social activities and FOF. Social activity focused on changes in social involvement from baseline to follow-up. Findings derived from Cox proportional-hazards models showed that a reduction in social activities was linked to a higher hazard of acquiring FOF across an 11-year follow-up period (---; HR = 1.81, *p* = 0.002; [12]), without providing information on whether the assumption of proportionality inherent to Cox models was fulfilled. Another study did not show conclusive evidence for a decline in social activities and the odds of developing FOF after controlling for sociodemographic variables (oo; OR = 0.58; 95% CI [0.17; 1.98]; [35]). A third study suggested that more frequent social participation was linked to increased odds for FOF at follow-up while controlling for other psychosocial factors and sociodemographic variables (---; OR = 1.17; 95% CI [1.07; 1.28]; [78]).

Two studies examined the relationship between activity level and FOF, with activity level being operationalized as self-reported restrictions of activities within the last year*.* In one study, the effect estimate indicated a link between a decreased activity level and a higher relative risk for the onset of FOF (---; RR = 2.32; 95% CI [1.40; 3.07]; [19]). They also reported an effect estimate indicating a link between a reduced activity level and a higher risk of persistent FOF (--; RR = 1.60; 95% CI [1.31; 1.96]), but controlling for other psychosocial factors and demographic variables did not further support the findings from the bivariate model (ooo; RR = 1.17; 95% CI [0.94; 1.46]; [19]). Results from the second study likewise did not provide evidence for increased odds of developing FOF between reduced activity levels, adjusting for sociodemographic variables (oo; OR = 0.53; 95% CI [0.24; 1.17]; [35]).

In addition, two studies reported information on the link between social support and FOF. There was no significant direction for the odds of developing FOF depending on ‘having someone to count on’ (OR = 1.25; 95% CI [0.90; 1.74]) and ‘the amount of friends seen monthly’ (OR = 0.98; 95% CI [0.95; 1.02]), both controlling for sociodemographic variables and other psychosocial factors (ooo; [17]). In contrast, one effect estimate indicated that ‘the amount of family members seen monthly’ was associated with increased odds of FOF above and beyond other psychosocial factors and sociodemographic variables (+ + +; OR = 1.03; 95% CI [1.03; 1.06]; [17]). Assessing the role of emotional support derived from the question whether the participant had anyone to talk to about problems or to help with difficult decisions suggested that having no emotional support was associated with an increased risk for developing FOF at 1-year follow-up, without controlling for covariates (-; RR = 2.64; 95% CI [1.57; 4.41]; [44]).

Another study investigated the relationship between subjective age and the onset of FOF. For assessing subjective age, participants were asked to report what age they felt most of the time during the last month. As common in studies on felt age, a proportional discrepancy score was calculated ([felt age minus actual age]/actual age) to measure subjective age. The findings indicate that higher subjective age was associated with increased odds of developing FOF, after controlling for other psychosocial factors and sociodemographic variables (---; OR = 1.24; 95% CI [1.08; 1.42]; [22]).

One study examined the association between social cohesion and the onset of FOF. Social cohesion was measured using a questionnaire comprising three questions relating to the extent of cohesion and solidarity within the community. Higher social cohesion was found to be protective against developing FOF above and beyond other psychosocial factors and sociodemographic variables (+ + +; OR = 0.82; 95% CI [0.71; 0.95]; [78]).

The same study investigated the relationship between reciprocity and the onset of FOF. Reciprocity was measured using three questions to report the extent of support and care received from others, which was not associated with developing FOF after controlling for psychosocial factors and sociodemographic variables (ooo; OR = 1.05; 95% CI [0.62; 1.78]; [78]).

## Discussion

This systematic review is the first to examine the longitudinal association between psychosocial factors and fear of falling (FOF) in older adults. The study aimed at identifying (1) which psychosocial factors were longitudinally examined in relation to FOF, (2) which psychosocial factors were longitudinally associated with FOF, (3) the level of evidence for specific psychosocial factors contributing to FOF, and (4) the extent to which the association between the specific psychosocial factor and FOF demonstrated incremental validity beyond sociodemographic variables and other psychosocial factors.

A total of 16 studies with 30,724 participants could be included. Overall, 14 different psychosocial factors were examined across primary studies (i.e., depressive and anxiety symptoms, positive and negative affect, self-efficacy, symptom burden, social activity, social participation, activity level, subjective age, social and emotional support, social cohesion, reciprocity). The present studies pursued three distinct approaches to investigating FOF as an outcome by considering either the onset of FOF (*k* = 10), persistent FOF (*k* = 2), both the onset and persistent FOF (*k* = 2), or changes in FOF (*k* = 2) over time.

The evidence rating indicated associations between higher depressive symptoms, anxiety symptoms, negative affect, symptom burden, lower social activity, higher subjective age, and less emotional support with FOF – that is, a greater risk for the onset and the persistence of FOF. In contrast, the studies included suggested that positive affect, self-efficacy, social support, and social cohesion were associated with lower FOF – that is, a lower risk for the onset and persistence of FOF.

Depressive symptoms were the most often examined psychosocial factor, showing a higher risk for FOF or increased levels of FOF in individuals with more severe depressive symptoms. However, findings were heterogeneous across primary studies. While four effect estimates showed a longitudinal association of depressive symptoms with FOF above and beyond other psychosocial factors and sociodemographic variables, four effect estimates showed this association only when controlling for sociodemographic variables and became insignificant when other variables were included. Finally, one effect estimate suggested no longitudinal association between depressive symptoms and FOF. These longitudinal findings differ from the results of a large number of cross-sectional studies summarized in previous systematic reviews by MacKay et al. [41] and Xiong et al. [82]. MacKay et al. [41] found that 16 studies examined the association between depressive symptoms and FOF and reported a positive relationship. In addition, Xiong et al. [82] included 28 studies that analyzed the relationship between depression (*k* = *22),* depressive symptoms (*k* = 6), and FOF and found that this factor (along with anxiety) was the most common significant psychological risk factor for FOF.

Less clear is the relationship between anxiety symptoms and FOF, which was examined longitudinally by only two studies of which one found anxiety to be unrelated to the onset of FOF even without controlling for covariates [44]; the other found that anxiety symptoms were a significant independent predictor for FOF even after adjusting for depressive symptoms [40].

The weak longitudinal association between anxiety symptoms and the onset of FOF might be explained by the conceptual overlap between these factors and their similar characteristics, like anxious cognitions and avoidance behavior [66]. In contrast, many cross-sectional studies found a significant positive relationship between anxiety symptoms and FOF (e.g. [30, 53]), and furthermore, a systematic review by Dissanayaka et al. [18] characterized FOF as an atypical presentation of anxiety disorder in people with Parkinson’s disease.

Similarly, the association between low activity levels and FOF is less clear. Whereas one study observed no association between social activity or activity level and the onset of FOF when controlling for age and gender [35], another study suggested an association between poorer activity levels and onset of FOF, but not persistent FOF [19], the latter might be due to an already lowered activity level prior to the onset of FOF due to physical conditions or impaired balance. Finally, two individual findings should be emphasized as they could stimulate future studies in this regard. First, the finding that higher subjective age was longitudinally associated with higher FOF [22]. This is in line with previous studies that showed associations of subjective age with various health outcomes (for a meta-analysis, see Westerhof et al. [80]). Health problems such as poor subjective health or low physical performance could mediate the relationship between subjective age and FOF, as the findings of Fundenberger et al. [22] suggest. Since subjective age and subjective health are longitudinally interrelated [64], future studies should try to disentangle the role of subjective age as a potential predictor or mediator in the relation between health and FOF.

One study found a surprising association between increased family contact and higher odds of the onset of FOF [17]. This finding contradicts previous research on the positive effects of social support on physical, mental, and cognitive health [6, 62, 81] and warrants further investigation into the role of family support in FOF development to avoid bias resulting from insufficient evidence.

With respect to the third research question on the level of evidence for specific psychosocial factors, the extant literature on longitudinal relationships between psychosocial factors and FOF provides little evidence. As shown in the results, the relationship between depressive symptoms and FOF has been the most frequently investigated, but with inconsistent results. In contrast, only a small number of effect estimates were available for the other psychosocial factors. The quality of studies is further limited by risks of bias that range from moderate to high, which also limits the evidence of the findings.

Regarding the fourth research question, incremental validity beyond sociodemographic variables and other psychosocial factors was identified most frequently (seven effect estimates) for the association between depressive symptoms and FOF. Incremental validity was also identified for the relationship between anxiety symptoms, symptom burden, social activity, activity level, subjective age, and FOF. However, given the inconclusive nature of the findings due to the paucity of evidence, it is difficult to draw definitive conclusions from these findings. To modify future interventions to reduce FOF, these findings could indicate that it may be worthwhile to further investigate the link with depressive symptoms to include it in the evidence-based interventions.

## Limitations

The results of our review should be interpreted in light of their limitations. First, only 16 studies investigated longitudinal relationships between psychosocial factors and FOF in older adults and thus met the inclusion criteria. Due to the limited evidence, it is difficult to derive conclusions about the association of specific psychosocial factors and different FOF outcome types (e.g., onset, persistence, or changes of FOF over time).

Second, a rating scheme was used to compare the levels of evidence for the different psychosocial factors. This approach was chosen due to the large methodological heterogeneity between studies (i.e., number and types of psychosocial factors examined) that prohibited a meta-analysis based on odds ratios. In accordance with recommendations of the Cochrane Collaboration [9], a vote counting approach was chosen as main evaluation scheme, supplemented by information from significance tests [34]. Nevertheless, this approach is subject to limitations inherent in statistical significance testing [2]. Consequently, as soon as a sufficient number of studies with greater methodological homogeneity exists, future systematic reviews should employ meta-analyses to derive meta-analytic effect estimates, thereby allowing for estimations of effect sizes. The inability to perform meta-analyses also precluded the use of standard methods for assessing potential publication bias [14]. Yet in some of the primary studies, the predictive value of the psychosocial factor was not the main focus of the research, which limits the potential impact of publication bias. Conversely, there was a large number of significant but small effect estimates, potentially indicating the presence of a publication bias.

Third, further limitations result from characteristics of the primary studies included and their low to moderate quality. An important component of the quality of longitudinal studies is the documentation of losses to follow-up. In our body of evidence, this has only been reported in three studies, with only one study providing information on differences between responders and those who were lost to follow-up. This lack of information may lead to substantially biased results. Furthermore, the absence of power analysis in the respective studies may have limited the validity and generalizability of the results. Finally, in research on FOF, the Falls Efficacy Scale [70] is considered a reliable and valid measure of FOF. However, it was only used in three of the overall 16 studies to assess FOF, while the remaining studies used one or two self-developed questions, which could have compromised the validity of the results.

## Implication for future research

The current systematic review showed that there is a limited body of research on the longitudinal relationship between psychosocial factors and FOF. Previous systematic reviews [41, 82] based on cross-sectional studies have focused on the associations of FOF with sociodemographic factors, health-related factors, and physical performance, while psychosocial factors have been neglected in primary studies.

Therefore, further longitudinal research is required in the area of psychosocial factors. This review has illustrated that longitudinal associations with FOF have not been examined for a large number of psychosocial factors. Studies based on cross-sectional analyses suggested, for example, that higher levels of FOF are also linked with perceived stress and loneliness, and that lower levels of FOF are related to higher levels of life satisfaction, optimism, self-efficacy, self-esteem, and self-regulation [25]. However, corresponding evidence on longitudinal associations is still lacking for those factors. This could be critical, as longitudinal links in particular could motivate prevention measures.

The present studies primarily investigated longitudinal associations, and models ought to be validated by prediction studies in a subsequent step. Since significant associations between variables do not connote high predictivity [39], it cannot be ruled out that models from the present studies yield associations with limited value for prediction. This problem is widespread since studies with a descriptive or hypothesis-testing approach inherently do not draw on methods to validate the models at hand [84]. Yet as we see it, there is a need for further research to address predictive questions regarding FOF and to integrate appropriate methods from the field of prediction studies. As for the latter, cross-validation offers a frequently used technique in prediction and machine learning, allowing one to estimate the average predictive performance of fitted models by partitioning data into multiple training and testing datasets [84]. In general, following a predictive analysis approach would tend to enhance the investigation of which factors adequately predict an increased risk or future risk of FOF rather than merely focusing on associations.

It has to be emphasized that causal explanation and prediction are distinct concepts that require different statistical approaches. Nevertheless, rigorous terminology bears implications for both prediction and explanation. Thus, when investigating onset, persistence, or change of FOF, it should be noted that the aforementioned terms represent broad concepts which need to be defined more narrowly based on measures of occurrence to which they refer, e.g., incidence or prevalence.

When aiming to explain the onset of FOF, incidence or incidence rates of FOF are better suited to explain potential causal pathways for FOF. Hence, further research should seek to address more precisely which factors play a role in the development of FOF by using established terminology and its underlying concepts. Incidence here is referred to as “the number of new cases […] divided by the person-time over the period” [24], p. 34). Further clarifying the role of potential risk factors for incidental FOF should be accompanied by robust methodology in design and analysis. The potential impact of psychosocial or other exposures for incidental FOF ideally needs to be modeled in a longitudinal design, e.g., by comparing rate ratios depending on the exposures of interest [65]. In order to be able to compare such estimates in a standardized way, it is necessary to assess the time at-risk [63]. Following our review findings, focusing on the role of depressive symptoms for incidental FOF may be of added value for further research.

Additionally, risk differences or adjusted attributable risk could also be used to estimate whether and what additional relative proportion of the prevalence of fear of falling can be explained by specific binary exposures [45].

The FES-I questionnaire offers a good instrument to monitor changes in fear of falling across time without arbitrarily categorizing subjects into groups, leaving as much information as possible, allowing one to further explain and predict possible changes in the FES-I score with the aforementioned factors. Subsequently, this may facilitate deriving candidate features for interventions that are modifiable and may help to reduce fear of falling.

We have identified other key methodological aspects that should be considered in future research. For example, the handling of missing data was often not sufficiently taken into account across the studies included and should be addressed adequately when analyzing data, since analysis of complete cases or listwise deletion is a major risk of bias when data is not missing completely at random [58]. Appropriate methods, such as multiple imputation, are available for this purpose and have gained widespread popularity when dealing with missing data [73].

When communicating results, odds ratios frequently have been interpreted as risk ratios which is a common problem. Yet, odds ratios will approximate the risk ratio only if the rare disease assumption is met [61]. For FOF, a wide range of prevalence proportions (6.96–90.34%) has been reported [82], and the chance that calculated odds ratios from samples with a high prevalence of FOF may overestimate the true underlying risk ratio cannot be ruled out. Finally, it should be noted that possible confounding was hardly dealt with in the design and analysis, nor were confounding and possible implications for results frequently discussed in the limitation sections of the studies included in this review. Confounding is an inherent problem in observational designs and should already be addressed when developing questions and research designs and within analyses [26, 74].

## Implications for the implementation of interventions

The extant findings concerning the relationships between psychosocial factors and FOF demonstrate that there is not yet sufficient evidence to derive recommendations for interventions. In order to optimize existing interventions to reduce FOF by addressing psychosocial factors, further research is needed to understand the pathways by which psychosocial factors may impact FOF outcomes.

Nevertheless, analyses focusing on study and intervention characteristics offer preliminary indications of factors associated with reduced FOF. A meta-analysis by Kruisbrink et al. [33] examined characteristics of randomized controlled trials (RCTs) and identified intervention characteristics that were associated with larger reductions in FOF. Specifically, interventions implemented within community-based settings, integrating exercise components, and those supervised by trained instructors (e.g., Tai Chi) have been associated with larger favorable effects on FOF.

A Cochrane review by Lenouvel et al. [36] examined whether cognitive behavioral therapy (CBT), with and without exercise, was more effective than care as usual or other control conditions in reducing FOF among community-dwelling older adults. The CBT-based intervention appears to reduce FOF, with effects observed post-intervention and possibly sustained. However, the overall certainty of evidence was low to moderate, due to the absence of blinding in the included RCTs and substantial heterogeneity in the measurement and operationalization of FOF, which limited the comparability and interpretability of outcome measures. Consequently, conclusions regarding the effectiveness of CBT in reducing FOF must be drawn with caution. The Cochrane review also highlights what was a limitation in our systematic review, namely the inconsistent measurement and operationalization of FOF.

## Conclusion

This systematic review showed high between-study heterogeneity in terms of study samples, FOF measures, psychosocial factors and FOF outcomes. Furthermore, studies reported different results, even if they examined the same psychosocial factor. Future research should therefore provide more evidence on the relationship between psychosocial factors and FOF, examining a broader range of psychosocial factors (e.g., loneliness, views on aging, perceived stress, self-esteem), using standardized instruments for the assessment of FOF (e.g., the FES-I). Such studies should particularly focus on mediating psychological, physiological, and behavioral mechanisms linking psychosocial factors with FOF.

## Supplementary Information

Supplementary Material 1.

Supplementary Material 2.

Supplementary Material 3.

## Data Availability

The material used for this review, which consists of the data extracted from included studies and the modified NOS, is provided in the Open Science Framework (OSF): (https:/osf.io/fptxw).

## Abbreviations

BMI: Body-Mass-Index
C: Changes in Fear of Falling
CI: Confidence Interval
E.g.: Exempli Gratia
Et al.: Et Alia
FES-I: Falls-Efficacy Scale International
FES- BR: Falls-Efficacy Scale Brazil
Fig: Figure
FOF: Fear of Falling
HR: Hazard Ratio
I.e.: Id Est
NOS: Newcastle Ottawa-Scale
O: Onset of Fear of Falling
OR: Odds Ratio
P: *P*- Value
P: Persistence of Fear of Falling
RR: Risk Ratio
RRR: Relative Risk Ratio
β: Beta

## Footnotes

1 For the purpose of this review, we use the terms predictor or predict to refer to an independent variable in regression models as this is commonly done in psychological research. The term does not imply a causal relationship between the psychosocial factor and the outcome fear of falling.

2 (OR = 1.02, 95% CI [1.03; 1.04]; [17]); (OR = 1.10; 95% CI [1.04; 1.16]; [42]); (HR = 1.16 95% CI [1.07; 1.26]; [47]); (OR = 1.11; 95% CI [1.04; 1.15]; [72]).

3 (HR = 1.24, p =.000); [12]); (OR = 1.77, 95% CI [1.21; 2.59]); [13]).

4 (OR = 3.54, 95% CI [1.23; 10.1]; [21]); (RR = 1.50, 95% CI [1.08; 2.08]; [19]); (HR = 1.45 95% CI [1.21; 1.74] [47]).
