# Supplementary material for: Longitudinal associations of psychosocial factors and fear of falling in older adults: a systematic review
Source: BMC Geriatr. 2026 Apr 29;26:610. doi: 10.1186/s12877-026-07463-1 (PMC13130717; doi:10.1186/s12877-026-07463-1)
Supplement: Supplementary file 1 — Supplementary Material 1. [file 12877_2026_7463_MOESM1_ESM.docx]

**Differences between protocol and final review**

|  | Protocol | Final review |
| --- | --- | --- |
| Review Team | No changes | |
| Theoretical background | No changes | |
| Review question | Which psychosocial factors are longitudinally associated with FoF in older adults? | Three more questions were added:  1. Which psychosocial factors were longitudinally examined in relation to FOF?  2. What is the level of evidence for specific factors contributing to FOF in older adults?  3. To what extent do association between the specific factor and FOF demonstrate incremental validity? |
| Eligibility criteria | Outcome: Fear of falling (FoF) as measured using standardized psychometric assessment tools (e.g., Falls Efficacy Scale International) | We included also studies using one and three self-developed questions, e.g., 'How afraid are you of falling?' to measure FOF. This has been done, because of the 16 studis involved, only three used the Falls Efficacy Scale. |
| Search strategy | No changes | |
| Data extraction (Selecting and coding) | No changes | |
| Data analyses | No changes | |
| Data synthesis |  |  |
