# Supplementary material for: Longitudinal associations of psychosocial factors and fear of falling in older adults: a systematic review
Source: BMC Geriatr. 2026 Apr 29;26:610. doi: 10.1186/s12877-026-07463-1 (PMC13130717; doi:10.1186/s12877-026-07463-1)
Supplement: Supplementary file 2 — Supplementary Material 2. [file 12877_2026_7463_MOESM2_ESM.docx]

**Search Strings**

**Embase.com including PubMed and Medline**

| # | Query |
| --- | --- |
| 1 | 'old* adult*':ab,ti OR 'old* person*':ab,ti OR 'old* people':ab,ti OR 'old* m?n':ab,ti OR 'old* wom?n':ab,ti OR elder*:ab,ti OR ag*ing:ab,ti OR geriatric:ab,ti OR senior*:ab,ti |
| 2 | 'senescence'/exp OR 'aged'/exp OR 'older adults'/exp OR 'aging'/exp OR 'geriatrics'/exp |
| 3 | #1 OR #2 |
| 4 | fear:ti,ab AND fall*:ti,ab OR 'fof':ti,ab OR ('self efficacy':ti,ab AND fall:ti,ab) OR (fall*:ti,ab AND anxiety:ti,ab) |
| 5 | 'fear of falling'/exp |
| 6 | #4 OR #5 |
| 7 | factor*:ti,ab OR correlat*:ti,ab OR predict*:ti,ab OR risk:ti,ab OR protect*:ti,ab |
| 8 | 'longitudinal' OR 'prospective' OR 'long-term' OR 'cohort study' OR 'panel' |
| 9 | 'longitudinal study'/exp OR 'prospective study'/exp OR 'cohort analysis'/exp OR 'panel study'/exp |
| 10 | #8 OR #9 |
| 11 | #3 AND #6 AND #7 AND #10 |

**APA PsycInfo via EBSCO Host (including PsycInfo and PsycArticles)**

| # | Query |
| --- | --- |
| 1 | (Title: old* AND Title: adult*) OR (Title: old* AND Title: person*) OR (Title: old* AND Title: people) OR (Title: old AND Title: m?n) OR (Title: old AND Title: wom?n) OR Title: elder* OR Title: ag*ing OR Title: geriatric OR Title: senior* OR (Abstract: old* AND Abstract: adult*) OR (Abstract: old* AND Abstract: person*) OR (Abstract: old* AND Abstract: people) OR (Abstract: old AND Abstract: m?n) OR (Abstract: old AND Abstract: wom?n) OR Abstract: elder* OR Abstract: ag*ing OR Abstract: geriatric OR Abstract: senior* OR (Keywords: old* AND Keywords: adult*) OR (Keywords: old* AND Keywords: person*) OR (Keywords: old* AND Keywords: people) OR (Keywords: old AND Keywords: m?n) OR (Keywords: old AND Keywords: wom?n) OR Keywords: elder* OR Keywords: ag*ing OR Keywords: geriatric OR Keywords: senior* |
| 2 | (Title: fear* AND Title: falling) OR Title: "fof" OR (Title: fall* AND Title: "self-efficacy") OR (Title: fall* AND Title: anxiety) OR (Abstract: fear* AND Abstract: falling) OR Abstract: "fof" OR (Abstract: fall* AND Abstract: "self-efficacy") OR (Abstract: fall* AND Abstract: anxiety) OR (Keywords: fear* AND Keywords: falling) OR Keywords: "fof" OR (Keywords: fall* AND Keywords: "self-efficacy") OR (Keywords: fall* AND Keywords: anxiety) |
| 3 | Title: factor* OR Title: correlat* OR Title: predict* OR Title: risk OR Title: protect* OR Abstract: factor* OR Abstract: correlat* OR Abstract: predict* OR Abstract: risk OR Abstract: protect* OR Keywords: factor* OR Keywords: correlat* OR Keywords: predict* OR Keywords: risk OR Keywords: protect* |
| 4 | Title: "longitudinal" OR Title: "prospective" OR Title: "long-term" OR Title: "cohort study" OR Title: panel OR Abstract: "longitudinal" OR Abstract: "prospective" OR Abstract: "long-term" OR Abstract: "cohort study" OR Abstract: panel OR Keywords: "longitudinal" OR Keywords: "prospective" OR Keywords: "long-term" OR Keywords: "cohort study" OR Keywords: panel |
| 5 | #1 AND #2 AND #3 AND #4 |
| Full search string | ((title: ("longitudinal") OR title: ("prospective") OR title: ("long-term") OR title: ("cohort study") OR title: (panel)) OR (abstract: ("longitudinal") OR abstract: ("prospective") OR abstract: ("long-term") OR abstract: ("cohort study") OR abstract: (panel)) OR (Keywords: ("longitudinal") OR Keywords: ("prospective") OR Keywords: ("long-term") OR Keywords: ("cohort study") OR Keywords: (panel))) AND ((title: (factor*) OR title: (correlat*) OR title: (predict*) OR title: (risk) OR title: (protect*)) OR (abstract: (factor*) OR abstract: (correlat*) OR abstract: (predict*) OR abstract: (risk) OR abstract: (protect*)) OR (Keywords: (factor*) OR Keywords: (correlat*) OR Keywords: (predict*) OR Keywords: (risk) OR Keywords: (protect*))) AND ((title: (fear*) AND title: (falling)) OR title: ("fof") OR (title: (fall*) AND title: ("self-efficacy")) OR (title: (fall*) AND title: (anxiety)) OR (abstract: (fear*) AND abstract: (falling)) OR abstract: ("fof") OR (abstract: (fall*) AND abstract: ("self-efficacy")) OR (abstract: (fall*) AND abstract: (anxiety)) OR (Keywords: (fear*) AND Keywords: (falling)) OR Keywords: ("fof") OR (Keywords: (fall*) AND Keywords: ("self-efficacy")) OR (Keywords: (fall*) AND Keywords: (anxiety))) AND ((title: (old*) AND title: (adult*)) OR (title: (old*) AND title: (person*)) OR (title: (old*) AND title: (people)) OR (title: (old) AND title: (m?n)) OR (title: (old) AND title: (wom?n)) OR title: (elder*) OR title: (ag*ing) OR title: (geriatric) OR title: (senior*) OR (abstract: (old*) AND abstract: (adult*)) OR (abstract: (old*) AND abstract: (person*)) OR (abstract: (old*) AND abstract: (people)) OR (abstract: (old) AND abstract: (m?n)) OR (abstract: (old) AND abstract: (wom?n)) OR abstract: (elder*) OR abstract: (ag*ing) OR abstract: (geriatric) OR abstract: (senior*) OR (Keywords: (old*) AND Keywords: (adult*)) OR (Keywords: (old*) AND Keywords: (person*)) OR (Keywords: (old*) AND Keywords: (people)) OR (Keywords: (old) AND Keywords: (m?n)) OR (Keywords: (old) AND Keywords: (wom?n)) OR Keywords: (elder*) OR Keywords: (ag*ing) OR Keywords: (geriatric) OR Keywords: (senior*)) |

**CINAHL**

| # | Query |
| --- | --- |
| 1 | TI ( "old* adult*" OR "old* person*" OR "old* people" OR "old m*n" OR "old wom*n" OR elder* OR ag*ing OR geriatric OR senior* ) OR AB ( "old* adult*" OR "old* person*" OR "old* people" OR "old m*n" OR "old wom*n" OR elder* OR ag*ing OR geriatric OR senior* ) OR SU ( "old* adult*" OR "old* person*" OR "old* people" OR "old m*n" OR "old wom*n" OR elder* OR ag*ing OR geriatric OR senior* ) |
| 2 | TI ( (fear AND fall*) OR "fof" OR (fall* AND "self-efficacy") OR (fall* AND anxiety) ) OR AB ( (fear AND fall*) OR "fof" OR (fall* AND "self-efficacy") OR (fall* AND anxiety) ) OR SU ( (fear AND fall*) OR "fof" OR (fall* AND "self-efficacy") OR (fall* AND anxiety) ) |
| 3 | TI ( factor* OR correlat* OR predict* OR risk* OR protect* ) OR AB ( factor* OR correlat* OR predict* OR risk* OR protect* ) OR SU ( factor* OR correlat* OR predict* OR risk* OR protect* ) |
| 4 | TI ( 'longitudinal' OR 'prospective' OR 'long-term' OR 'cohort study' OR 'panel' ) OR AB ( 'longitudinal' OR 'prospective' OR 'long-term' OR 'cohort study' OR 'panel' ) OR SU ( 'longitudinal' OR 'prospective' OR 'long-term' OR 'cohort study' OR 'panel' ) |
| 7 | (TI 'longitudinal' OR 'prospective' OR 'long-term' OR 'cohort study' OR 'panel' OR AB 'longitudinal' OR 'prospective' OR 'long-term' OR 'cohort study' OR 'panel' OR SU 'longitudinal' OR 'prospective' OR 'long-term' OR 'cohort study' OR 'panel') AND (S1 AND S2 AND S3 AND S4) |

**Cochrane Library**

| # | Query |
| --- | --- |
| 1 | (old* NEXT adult*) OR (old* NEXT person*) OR (old* NEXT people) OR (old NEXT m?n) OR (old NEXT wom?n) OR elder* OR ag*ing OR geriatric OR senior* |
| 2 | (fear* NEXT falling) OR "fof" OR (fall* NEXT "self-efficacy") OR (fall* NEXT anxiety) |
| 3 | factor* OR correlat* OR predict* OR risk OR protect* |
| 4 | "longitudinal" OR "prospective" OR "long-term" OR "cohort study" OR panel |
| 5 | #1 AND #2 AND #3 AND #4 |

**Scopus.com**

| # | Query |
| --- | --- |
| 1 | ( old* AND adult* ) OR ( old* AND person* ) OR ( old* AND people ) OR ( old AND m?n ) OR ( old AND wom?n ) OR elder* OR ag*ing OR geriatric OR senior* |
| 2 | ( fear* AND falling ) OR "fof" OR ( fall* AND "self-efficacy" ) OR ( fall* AND anxiety) |
| 3 | factor* OR correlat* OR predict* OR risk OR protect* |
| 4 | "longitudinal" OR "prospective" OR "long-term" OR "cohort study" OR panel |
| 5 | #1 AND #2 AND #3 AND #4 |
| Full search string | ( TITLE-ABS-KEY ( ( old* AND adult* ) OR ( old* AND person* ) OR ( old* AND people ) OR ( old AND m?n ) OR ( old AND wom?n ) OR elder* OR ag*ing OR geriatric OR senior* ) AND TITLE-ABS-KEY ( ( fear* AND falling ) OR "fof" OR ( fall* AND "self-efficacy" ) OR ( fall* AND anxiety ) ) AND TITLE-ABS-KEY ( factor* OR correlat* OR predict* OR risk OR protect* ) AND TITLE-ABS-KEY ( "longitudinal" OR "prospective" OR "long-term" OR "cohort study" OR panel ) ) |

**Web of Science**

| # | Query |
| --- | --- |
| 1 | ( old* AND adult* ) OR ( old* AND person* ) OR ( old* AND people ) OR ( old AND m?n ) OR ( old AND wom?n ) OR elder* OR ag*ing OR geriatric OR senior* |
|  | ( fear* AND falling ) OR "fof" OR ( fall* AND "self-efficacy" ) OR ( fall* AND anxiety) |
| 2 | factor* OR correlat* OR predict* OR risk OR protect* |
| 4 | "longitudinal" OR "prospective" OR "long-term" OR "cohort study" OR panel |
| 5 | #1 AND #2 AND #3 AND #4 |
| Full search string | ( old* AND adult* ) OR ( old* AND person* ) OR ( old* AND people ) OR ( old AND m?n ) OR ( old AND wom?n ) OR elder* OR ag*ing OR geriatric OR senior* (Topic) and ( fear* AND falling ) OR "fof" OR ( fall* AND "self-efficacy" ) OR ( fall* AND anxiety) (Topic) and factor* OR correlat* OR predict* OR risk OR protect* (Topic) and "longitudinal" OR "prospective" OR "long-term" OR "cohort study" OR panel (Topic) |
