# Supplementary material for: Longitudinal associations of psychosocial factors and fear of falling in older adults: a systematic review
Source: BMC Geriatr. 2026 Apr 29;26:610. doi: 10.1186/s12877-026-07463-1 (PMC13130717; doi:10.1186/s12877-026-07463-1)
Supplement: Supplementary file 3 — Supplementary Material 3. [file 12877_2026_7463_MOESM3_ESM.docx]

**Supplementary Material 3**

Measurement instruments and response formats used to assess fear of falling (FOF)

| Study | Measurement instrument / item(s) | Number of items | Response format | Operationalization / categorization of FOF |
| --- | --- | --- | --- | --- |
| Austin et al., 2007 | Three self-developed questions:  "Are you afraid of falling?"  "Do you limit any any household activities because you are frightened you may fall?" "Do you limit any outside activities because you are frightened you may fall?" | 3 | Binary (yes/no) | One positive response classified as FOF |
| Clemson et al., 2015 | Single global FOF question:  NR | 1 | 4-point Likert (not at all afraid – very afraid) | Higher categories indicate greater FOF |
| Curcio et al., 2020 | Falls Efficacy Scale–International (FES-I) | 16 | 4-point Likert (1 = not at all concerned to 4 = very concerned) | Sum score (16–64); categorized into no/low, moderate, high FOF |
| Dierking et al., 2016 | Single FOF question: “How afraid are you of falling?” | 1 | 4-point Likert (not afraid – very afraid) | Higher responses reflect greater FOF |
| Drummond et al., 2020 | Falls Efficacy Scale–International – Brazilian version (FES-I-BR) | 16 | 4-point Likert | Sum score (16–64); dichotomized (≥23 = FOF) |
| Freiberger et al., 2022 | Single FOF question  NR | 1 | 4-point Likert (not at all concerned – very concerned) | Categorized as absent, transient, or persistent FOF |
| Fundenberger et al., 2022 | Single FOF question: “Did you worry about falling?” | 1 | Binary (yes/no) | Yes classified as FOF |
| Lach et al., 2005 | Single FOF question:  “At the present time are you very fearful, somewhat fearful, or not fearful that you might fall (fall again)?" | 1 | 3-point ordinal scale (not fearful – very fearful) | Higher category indicates greater FOF |
| Lindh Renghifo et al., 2019 | Falls Efficacy Scale–International (FES-I) + single FOF question “Are you afraid of falling?” | 16 + 1 | 4-point Likert (FES-I); binary (yes/no) | FES-I sum score; additional dichotomous FOF measure |
| Luo et al., 2022 | Two questions: “In the last month, did you worry about falling down?”  “In the last month, did this worry ever limit your activities?” | 2 | Binary (yes/no) | Three-category variable: no FOF; FOF without restriction; FOF with restriction |
| Makino et al., 2021 | Single FOF question: “Are you afraid of falling?” | 1 | 4 response options (ordinal) | Higher responses indicate FOF |
| Murphy et al., 2003 | Single FOF question: “Are you afraid of falling?” | 1 | Binary (yes/no) | Yes classified as FOF |
| Oh Park et al., 2011 | Single FOF question: “Did you have fear of falling in the last 2 months or since the last interview?” | 1 | Binary (yes/no) | Yes classified as FOF |
| Peng et al., 2024 | Two questions:  “Did you worry about falling”; and “Did this worry ever limit your activities” over the last month. | 4 | Binary (yes/no) | Separate binary indicators for FOF and FOF-related restriction |
| Uemura et al., 2015 | Single FOF question: “Are you afraid of falling?” | 1 | 4 response options | Dichotomized into fear vs. no fear |
| Wang et al., 2023 | Single FOF question “Are you very worried about falls?” | 1 | Binary (yes/no) | Yes classified as FOF |
